# A global dataset of key traits for plant belowground functioning

**DOI:** 10.1038/s41597-026-08151-w

**Published:** 2026-08-29

**Authors:** Helge Bruelheide, Karl Andraczek, Aline B. Bombo, Gabriella Damasceno, Ying Fan, Grégoire T. Freschet, Lena Hartmann, Justus Hennecke, Cody Coyotee Howard, Saheed Olaide Jimoh, Jitka Klimešová, Daniel C. Laughlin, Liesje Mommer, Tsumbedzo Ramalevha, Frances Siebert, Shersingh Joseph Tumber-Dávila, Alexandra Weigelt, David Atkins, Gianmaria Bonari, Trevor A. Carter, Jiří Doležal, Timothy Harris, Borja Jiménez-Alfaro, Austin R. Kelly, F. Curtis Lubbe, Jana Martínková, Mathieu Millan, Jacqueline Ott, Steve Orzell, Jianqiang Qian, Alice E. Stears, Dennis Whigham, Xin Jing, Joana Bergmann, Alessandra Fidelis

**Affiliations:** 1 Institute of Biology/Geobotany and Botanical Garden, Martin Luther University Halle-Wittenberg, Am Kirchtor 1, 06108 Halle, Germany; 2 German Centre for Integrative Biodiversity Research (iDiv) Halle-Jena-Leipzig, Puschstr. 4, 04103 Leipzig, Germany; 3 Systematic Botany and Functional Biodiversity, Institute of Biology, Leipzig University, Leipzig, Germany; 4 Leibniz Centre for Agricultural Landscape Research (ZALF), Müncheberg, Germany; 5 Lab of Vegetation Ecology, Instituto de Biociências, Universidade Estadual Paulista (UNESP), Rio Claro, Brazil; 6 Department of Earth and Planetary Sciences, Rutgers University, Piscataway, NJ USA; 7 Theoretical and Experimental Ecology Station, CNRS, University of Toulouse III, Moulis, France; 8 Richard-Muth-Platz 6, 14552 Michendorf, Germany; 9 Forest Ecology and Forest Management Group, Wageningen University, Wageningen, The Netherlands; 10 Department of Biology, University of Central Florida, Orlando, Florida USA; 11 Department of Botany, University of Wyoming, Laramie, Wyoming, USA; 12 Department of Bioscience, Rice University, Houston, TX 77005 USA; 13 Institute of Botany of the Czech Academy of Sciences, Třeboň, Czech Republic; 14 Unit for Environmental Sciences and Management, North West University, Potchefstroom, South Africa; 15 South African Environmental Observation Network (SAEON), Pretoria, South Africa; 16 Expanded Freshwater & Terrestrial Environmental Observation Network, Pretoria, South Africa; 17 Department of Environmental Studies, Dartmouth College, Hanover, New Hampshire USA; 18 Department of Life Sciences, University of Siena, Siena, Italy; 19 National Biodiversity Future Center (NBFC), Palermo, Italy; 20 Department of Forest and Rangeland Stewardship, Colorado State University, Fort Collins, CO USA; 21 Royal Botanic Gardens, Kew, London, United Kingdom; 22 Biodiversity Research Institute (IMIB), University of Oviedo–CSIC–Principality of Asturias, Mieres, Asturias 33600 Spain; 23 Department of Organismal and Systems Biology, University of Oviedo, Oviedo, Asturias 33071 Spain; 24 ARK Ecological Consulting, Sidney, Texas USA; 25 Department of Ecology and Conservation Biology, Texas A&M University, College Station, Texas USA; 26 S.M. Tracy Herbarium, Texas A&M University, College Station, TX USA; 27 Université de Mayotte, Dembéni, France; 28 AMAP, Université de Montpellier, CIRAD, CNRS, INRAE, IRD, Montpellier, France; 29 USDA Forest Service, Rocky Mountain Research Station, Forest and Grassland Research Laboratory, Rapid City, SD USA; 30 Avon Park Air Force Range, Avon Park, FL USA; 31 College of Forestry, Henan Agricultural University, Zhengzhou, China; 32 Center For Adaptable Western Landscapes, Northern Arizona University, Flagstaff, AZ USA; 33 Smithsonian Environmental Research Center, Edgewater, MD USA; 34 State Key Laboratory of Herbage Improvement and Grassland Agro-Ecosystems, and College of Pastoral Agriculture Science and Technology, Lanzhou University, Lanzhou, Gansu China

## Abstract

Belowground functional diversity is relevant to numerous ecosystem functions and to ecosystem resilience under global change, yet quantitative information on belowground plant traits is disparate and poorly integrated across fields of research. So far, belowground plant traits have been studied in three disparate research domains: (i) fine root traits in the context of belowground resource economics and symbioses; (ii) maximum rooting depth and lateral extent of the root system in the context of overall plant allometry and resource uptake; (iii) clonal organs and bud banks with a focus on plant and community resilience. However, there remains a major disconnection among these three fields of research. A prerequisite for linking them together is the creation of a comprehensive, curated, open-access dataset available for comprehensive analyses. Here, we compiled and harmonised such a trait dataset for 10,453 vascular plant species and 19 belowground plant traits, which we named UNDERPLOT. Based on these data, we defined two integrative indices, a Belowground Persistence Type (BPT) and a Clonal Spread Index (CSI), which are suitable trait-based indicators for predicting resistance and resilience of communities to disturbance. Further applications will increase our knowledge of the dimensionality of the belowground trait space, adding new insights into trait–environment relationships, vegetation responses to climate change and disturbance, as well as a deeper understanding of evolutionary first principles.

## Background & Summary

Our understanding of global biodiversity requires a comprehensive assessment of functional diversity, yet several key aspects of that diversity facet remain a mystery. Despite recent global-scale syntheses^1–3^, our knowledge of the variation of plant functional strategies remains both incomplete^4^ and, worse yet, lacks cohesion across the subdisciplines of functional biodiversity science. This has resulted in contradictory ontologies and inconsistent use of scientific terms, which we address below for belowground plant organs. In part, these inconsistencies stem from the legacies of how science unfolded in the global north and south, as well as from insufficient scientific communication between those regions^5^. Then, across ecosystems, different aspects of belowground plant traits were analysed. For example, the ability to resprout from root crowns, xylopodia and lignotubers has been a major research field in savannas disturbed by fire^6^, while rooting depth and lateral spread were analysed as responses to water availability^7^. Thus, trait data had been collected for ecosystem-specific questions or simply due to random ideas on different sites and were regionally restricted. This has resulted in records of phylogenetic and functional diversity that are clustered according to geopolitical boundaries, rather than according to true global patterns of diversity^5^. Cross-disciplinary integration is also difficult to achieve, given the additional challenge of unifying concepts across fields while simultaneously accommodating different perceptions and methodological developments at the global scale^8^. Nonetheless, synthesis science tailored to specific research needs is crucial for guiding research and public policies about biodiversity in all existing facets^9,10^. Although many functional traits of plants are well-studied, such as the leaf^11^ or root economics space^12^, research gaps remain.

A true integration of all plant belowground traits with more commonly studied aboveground traits is necessary to comprehensively characterize whole-plant trait diversity and the functions they provide. Therefore, a broader synthesis of available data on belowground plant traits is critical for understanding patterns in plant strategies and ecosystem functioning, and to predict vegetation responses to anthropogenically induced changes in global climate and disturbance regimes. Still, belowground plant functional traits have been less studied and are less frequently incorporated into global plant trait databases, likely due to difficulties in obtaining belowground material. Concerted efforts to unearth, examine, synthesize and analyse the contribution of plant belowground traits to ecosystem functioning have begun to uncover important relationships, for example, the role of belowground traits in enhancing ecosystem resilience to severe disturbance^6^. Our aim was (i) to put together the existing datasets to identity gaps, and (ii) to interpret some of regionally used traits in light of other functions, even when it was not primary interest of the original researchers.

Here, we present an integration of data on different belowground plant components, termed the UNDERPLOT (UNDERground PLant Organ Trait) dataset, assembled by teams working across different biomes, collecting plant trait data across various belowground organs. Thus, our dataset compiles information that can be used to understand and predict how vegetation dynamics are modulated by belowground organs. This dataset captures a wide spectrum of belowground organ types, morphologies and functions across diverse environmental gradients^13^. These divergent datasets were standardized into a comprehensive dataset that includes information on belowground plant traits, including fine-root traits, the extent of the whole root system in both horizontal and vertical dimensions, as well as clonal and bud bank traits. Although separate databases and analyses exist for belowground plant traits (i.e, GRooT^14^, RSIP^15^, CLO-PLA3^16^), here we not only combine and taxonomically harmonize these data but also expand them for more species. The resulting dataset contains 19 belowground plant traits and two calculated indices on belowground persistence and clonal spread for species that occur across a large (albeit clustered) geographical area.

The dataset will facilitate the integration of plant species’ belowground traits with aboveground traits, as, for example, those found in TRY^17^, thereby enabling a more complete assessment of the dimensionality of global plant form and function. Current theory assumes at least three global axes of plant strategies^3^: (1) the conservation gradient, being part of both the leaf economics spectrum (LES)^11^ and the root economics space (RES)^18^, reflecting the trade-off between cheaply constructed organs with fast resource acquisition and more durable organs with slow rates of return, (2) the collaboration gradient, reflecting the trade-off between resource acquisition with thin fine roots and thick fine roots with symbiosis with mycorrhizal fungi^12^, and (3) the plant-size gradient^2,19^, reflecting the trade-off between early reproduction and longevity^20^. The UNDERPLOT dataset integrates these gradients with clonality, rooting system and persistence to achieve a more holistic understanding of plant functioning. These data will enable deeper analysis of trait–environment relationships, which has already been underway for aboveground traits^21^, fine root traits^22^ and rooting depth^23–25^. Finally, the UNDERPLOT dataset could facilitate inference about relationships between plant traits and multiple aspects of ecosystem functioning^26,27^, such as aboveground productivity and its temporal stability, drought resistance, decomposition, carbon sequestration, nutrient cycling and other soil microbial processes, water storage, soil anchorage, persistence and regeneration under limited water availability, and post-disturbance recovery. Among other applications, it could also help identify sets of species or traits to meet restoration targets^28^, especially for non-forest plant communities^29^ where resilience to disturbance often plays an important role^30^.

## Methods

We compiled the data from 14 different sources (Tables 1, 2 and 3). Species names in each database were taxonomically standardized (see *Taxonomic standardization*), but the original taxonomic names were kept in separate columns. In this way, later access to additional information, such as traits not combined in UNDERPLOT is possible through original, edited, parsed, resolved and aggregated names. Information on the number of replicates per species was also included, enabling future analyses based on individuals. In a second step, we harmonized the names of clonal growth organs, life forms and defined the BPT and CSI as described below. In a third step, the datasets were combined, which involved calculating species mean values for numeric variables (Table 1) or collating different assessments of categorical variables (Tables 2 and 3) per aggregated species name.

**Table 1:** List of all numeric plant traits included in the UNDERPLOT dataset, grouped into traits related to fine roots, the overall root system or belowground clonality.

Category	Abbreviation	Trait	Unit	n	mean	Q 25	median	Q 75	Source
Fine root	RN	Root nitrogen concentration	mg/g	1841	13.66	8.31	12.10	17.50	^14,54^^,a^
	RD	Root diameter	mm	1836	0.42	0.23	0.35	0.51	^14,54^^,a,b^
	RTD	Root tissue density	g/cm^3^	1625	0.30	0.16	0.26	0.39	^14,54^^,a,b^
	SRL	Specific root length	m/g	2137	91.69	21.20	51.20	114.76	^14,54^^,a,b^
Root system	RDepth	Maximum rooting depth	m	2734	1.57	0.43	0.90	1.70	^15^
	LRExtent	Lateral rooting extent (maximum radius of the root system)	m	1797	1.57	0.25	0.48	1.11	^15^
	Coarse.fine.ratio	Coarse root to fine root mass ratio	g/g	134	11.07	1.75	5.00	12.89	^14^
Clonality	BBdepth	Bud bank depth	cm	2803	3.50	2.30	3.80	4.00	^16^^,c^
	BBsize	Bud bank size per shoot	total number of buds/shoot	3203	12.79	5.00	12.96	20.00	^16^^,a,c,d^
	n.offspring.per.shoot	Number of offspring shoots produced per parent shoot per year	number	1915	2.45	1.00	1.30	3.50	^16^^,c^
	Lateral.expansion	Lateral spread (distance increment between mother and daughter shoot per year)	cm/year	1964	7.33	0.50	4.70	13.00	^16^^,c^
	Connection.persistence	Persistence of connections between ramets	years	1950	3.53	2.40	4.00	4.00	^16^^,c^
	CloGenExt	Extent of the genet (maximum distance between the youngest shoot and the oldest attached part of the clonal growth organ) ^f^	cm*years	1906	24.41	2.00	13.00	35.20	^16^^,c^
Indices	is.woody.below	Noteworthy lignification of the belowground parts	logical	10132					^15,17,50,55,56^^,e^
	is.woody.above	Noteworthy lignification of the aboveground parts	logical	7583					^15,17,50,55,56^^,e^
	is.clonal	Ability to produce physically independent individual plants (vegetative reproduction)	logical	7306					^15,17,50,55,56^^,e^
	is.resprouting	Ability to resprout after total aboveground biomass loss	logical	2820					^15,17,50,55,56^
	BPT	Belowground Persistence Type	categorical	7323	3.36	2.00	3.00	5.10	see text and Fig. 1
	CSI	Clonal Spread Index	categorical	6564	0.63	0.00	0.00	1.00	see text and Fig. 2
Whole plant	CGO	Clonal growth organ	categorical	7214					see Table 2
	Growth form	Growth form unified from different data sources	categorical	8997					see Table 3

Number of species (n) shows the number of entries in the UNDERPLOT dataset. Q 25 and Q 75 show the 25th and 75th percentiles, respectively. The number in Sources refers to publications in the reference list, while letters refer to unpublished data described in the footnotes. With additional unpublished data provided by (a) USA grassland trait compilation, (b) Sandhill grassland traits, (c) CLO-PLA4 (https://clopla.butbn.cas.cz/), (d) Alessandra Fidelis, Aline B. Bombo and the Forb Ecology Research Group, RSA, (e) International Tundra Experiment (ITEX, https://www.gvsu.edu/itex/synthesis-data-4.htm), (f) CloGenExt calculated from^16^ as the product of Lateral.expansion and Connection.persistence.

**Table 2:** List of all categories of clonal growth organs (CGOs) or absence of CGOs (in case of annuals and non-clonal herbaceous or woody perennial species) included in UNDERPLOT.

Categories	Definition	BPT	n
annual	Life cycle completed in one year or less, no CGO	1	807
herbaceous perennial non-clonal	Non-woody, non-clonal plant with a life span of more than one year, usually with a perennial main root for the whole life	2	1300
bulb	Compressed belowground stem with storage leaves	3	153
clonal herb	Herbaceous plants capable of producing independent rooting units (ramets) during their lifespan	3	180
stem tuber	Thickened stems formed by rhizomes or stolons	3	73
no belowground roots	Without roots in the soil	3	33
root tuber	Thickened roots	3	46
stolon	Rooting horizontal stem aboveground or on the soil surface	3	178
rhizome	Belowground stem with adventitious roots, includes epigeogenous and hypogeogenous rhizomes, and those for which detailed information was not available	3, 6a, 6b	2095
non-woody rhizome	Rhizome without noteworthy lignification	3	12
epigeogenous rhizome	Rhizome initiated at the soil surface, bearing green leaves, with its final position belowground	3, 6a, 6b	999
hypogeogenous rhizome	Rhizome initiated belowground, bearing scale leaves, with its final position belowground	3, 6a, 6b	828
root sprouting	Ability to form buds on roots and sprout shoots from them spontaneously	3, 6a, 6b	202
woody non-clonal	Non-clonal, lignified plant	4	200
lignotuber	Tuberous, lignified thickening of the base of the shoot, originated from the activity of accessory buds at the cotyledonary node and axillary buds of upper stem nodes	5a, 5b	669
xylopodium	Lignified, swollen basis of smaller shrubs and forbs, a special case of the main root or formed by root and shoot together	5a, 5b	418
woody rhizome	Rhizome with noteworthy lignification	6a, 6b	249
main root	The primary main root, also including the root crown and hypocotyl tubers	1, 2, 3, 4, 5, 6	517
NA			3239

**Fig. 1:**
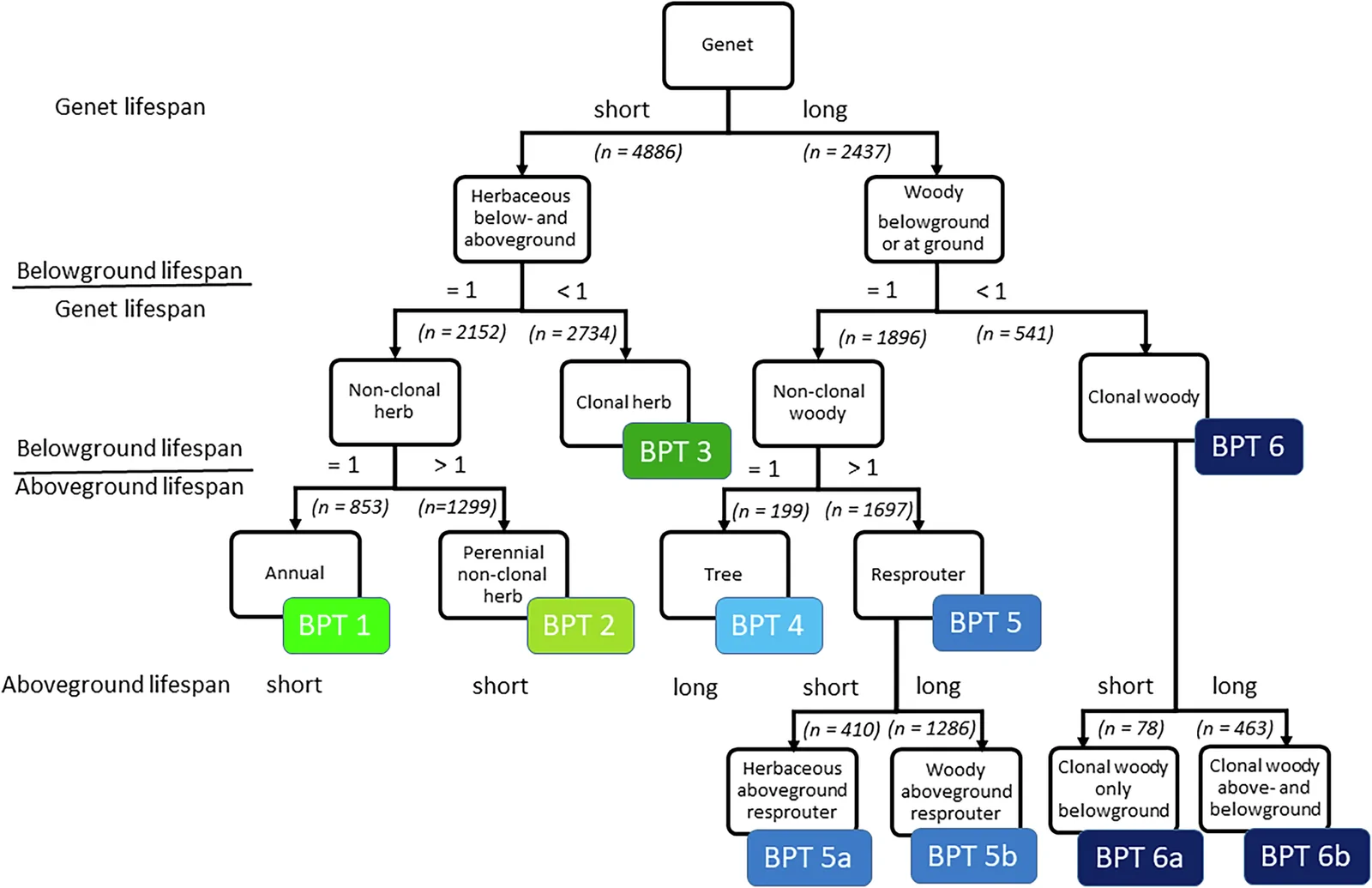
Decision tree defining the Belowground Persistence Type (BPT), following the concept of Klimešová *et al*.^38^, and *n* shows the number of taxa in each category.

Definitions follow Wagenitz^45^. BPT stands for Belowground Persistence Type according to Fig. 1, and n refers to the number of species with the respective trait. Note that the same species might be counted in more than one category. NA = neither clonal growth organ (CGO) nor absence of CGO reported. Data from^16,50,55,56^ with additional unpublished data provided by A. Fidelis, F. Siebert, T. Ramalevha, A. Bombo, J. Klimešová, and H. Bruelheide.

**Table 3:** Main categories of growth forms included in UNDERPLOT.

Categories	Definition	BPT	n
annual	Life cycle completed in one year or less	1	815
aquatic	Plants growing submersed	3	4
climber	Plants that climb up trees and other tall objects	2, 5, 6	33
clonal perennial	Clonal, perennial herbaceous plant	3, 6	3546
clonal woody	Clonal, woody plant	6a, 6b	367
epiphyte	Plant that grows on another (host) plant, without contact with the soil	—	1
fern	Vascular plant belonging to the class Polypodiopsida that reproduces via spores	—	23
forb	Non-graminoid, aboveground herbaceous, perennial, flowering plant	1, 2, 3, 5a, 6a	1687
graminoid	Herbaceous plant with a grass-like morphology	1, 2, 3	336
liana	Long-stemmed, woody vine	5a, 5b, 6a, 6b	25
non-clonal perennial	Non-clonal, perennial herbaceous plant	2, 4, 5a	1153
non-clonal woody	Non-clonal, woody plant	4, 5a,5b	244
palm	Perennial, flowering plants in the monocot family Arecaceae	4, 6b	1
shrub	Small-to-medium-sized plant with (often multiple) woody stems aboveground	4, 5b, 6b	1164
subshrub	Herbaceous above, woody at the base only	5a, 5b, 6a, 6b	219
tree	Woody plant with a distinct trunk and crown	4, 6a, 6b	1278
NA			1456

Definitions follow Wagenitz^45^. BPT stands for Belowground Persistence Type according to Fig. 1, and n refers to the number of species with the respective trait. Note that the same species might be counted in more than one category. When the information was available, the more detailed categorization was used, and when not, the species was assigned to the basic categories. NA = no growth form reported, - = not assigned to any BPT. Data from^16,17,50,55,56^ with additional unpublished data and classification provided by H. Bruelheide.

### Taxonomic standardization

The different datasets and new data we collated in UNDERPLOT came from different sources and, thus, did not follow a unified taxonomic reference. We used the standardized taxonomy of the sPlot 4.1 database, the newest version of the global database of vegetation plot records^31^, to integrate all taxonomic information under a single taxonomic backbone. This decision was made because the sPlot 4.1 backbone already provided the link to the latest version of TRY (version 6), the largest and most comprehensive plant trait database^17^. Using sPlot 4.1 also allowed us to combine traits from UNDERPLOT with species composition data from sPlot 4.1. The sPlot 4.1 taxonomic backbone is based on World Flora Online (WFO)^32^, which is an open-access, web-based compendium of the world’s plant species. We downloaded the WFO taxonomic backbone on November 15, 2025 (v. 2025.06, https://doi.org/10.5281/zenodo.15704590), which contained 1,648,360 names. This WFO version was used both for creating the sPlot 4.1 backbone as well as for new names in UNDERPLOT. First, we processed the original name (Original_name) by removing strange characters, double blanks and authority names (Edited_name). Then, the terms for subspecies, variants, and hybrids were standardized (Parsed_name) and run against the sPlot backbone, which contained 413,663 shared names between sPlot 4.1 and TRY v6. As a result, the present valid name was returned (Resolved_name). Species that were not found in the combined sPlot-TRY backbone were run directly against the WFO backbone, using the WorldFlora R package^33^, with standard settings and a maximum relative Levenshtein distance of 0.1. In addition, non-matching entries were inspected on an individual basis. To summarize all trait values at the taxonomic species level, subspecies and variants were aggregated (Aggregated_name). The resulting dataset contains one row per Aggregated_name.

### Traits

We included the most frequently measured fine root traits from GRooT^14^, complemented by further published sources^34^, which were root nitrogen concentration (RN), root diameter (RD), root tissue density (RTD) and specific root length (SRL). These root traits define the conservation-collaboration gradients in fine-root economics^12^. While these two gradients define the strategies for belowground resource acquisition^12^, they do not capture those for large-scale spatial exploration and persistence. Space exploration is reflected in rooting depth (RDepth), i.e., the maximum observed depth of roots^15^, and lateral rooting extent (LRExtent), i.e., the maximum one-sided (radius) horizontal distance from the stem reached by the roots^15^. Bud bank depth (BBdepth) and size (BBsize) were obtained from CLO-PLA3^16^. A list of all numeric traits included in UNDERPLOT is provided in Table 1. In addition to published data, we also included new, yet unpublished datasets (see footnote in Table 1). For species, for which we had fine root traits but lacked information on woodiness, resprouting capacity and clonality, we additionally conducted extensive searches to fill in this information (Supplementary Information S1). If we encountered conflicting information in different souces on the same species, we list all sources and then decided for the most plausible one (indicated with “1” in the “chosen” column in Supplementary Information S1). The number of belowground buds and their depth in the soil were assessed based on the assumption that each leaf (or leaf scale) axil contains a bud, and this number is recorded per shoot for clonal plants and per whole rooting unit for non-clonal plants^35^. A bud bank can provide a demographic storage effect stabilizing population dynamics, and also confer resistance to disturbance and invasion^36^. Bud bank depth gives the mean bud bank depth, calculated from bud bank numbers at different soil depths. Bud bank size reports the total number of buds across all soil depths. n.offspring.per.shoot is the number of offspring shoots produced per parent shoot per year in a clonal plant. Lateral spreading distance (Lateral.expansion) is only provided for clonal plants, excluding freely dispersible clonally formed organs that have separated from the mother plant soon after formation (e.g. bulbils), and refers to the increment of clonal growth in the horizontal direction from the maternal shoot^16^. In case of disrupted connections between ramets, the whole genetic individual may be much larger^37^. The reported values are means of all records for the given species. The persistence of a clonal growth organ (Connection.persistence) is the time span of the physical connection between mother and daughter shoots, and the numbers are only truncated values because morphological analysis only permits the estimation of persistence for a few years^16^. In CLO-PLA3, persistence is categorized into 0.5, 1.5 and 4 years, and the reported values are means of all records for a given species. Finally, we calculated the extent of physically connected parts of the genet (CloGenExt) by multiplying Lateral.expansion with Connection.persistence.

In addition to compiling numeric values for belowground plant traits, we also harmonized the names of clonal growth organs (Table 2) and growth forms (Table 3). In Table 2, we also included categories representing the absence of clonal growth organs (CGOs), such as annuals, non-clonal herbaceous perennial and woody plant species as well as the absence of roots. In addition, Table 2 contains organs that allow resprouting from belowground organs.

### Definition of Belowground Persistence Types (BPTs)

Based on the available traits in Table 1, most of the species in UNDERPLOT (i.e. 7,323 out of 10,453) could be assigned to Belowground Persistence Types (BPTs). Following the recent concept^38^, the degree of belowground persistence has been based on three attributes, which are hierarchically combined (Fig. 1): (1) woodiness, reflecting belowground longevity; (2) clonality, endowing plants with vegetative regeneration capability and the potential for spread and multiplication; (3) resprouting ability, allowing plants to replace aboveground shoots. Each of these three characteristics is available in Table 1 in binary form.

These traits confer on plant species two conceptually important functions: the ability to persist after disturbance and, in the case of clonal plants, the ability to spread laterally. Both persistence and spreading distance do not depend on a single particular morphological structure; they can be facilitated by different structures. It should also be pointed out that persistence here refers to individual plants, not to populations. For example, population persistence after disturbance can also occur through regeneration from seeds. Conversely, the belowground organs that confer persistence or spread might also serve further functions, such as anchorage or resource storage^13^.

We defined the Belowground Persistence Type (BPT) by making use of information about whether a species is woody below- or aboveground, clonal or can resprout (Fig. 1). The BPT is a categorical variable with ordinal properties that describes the ability of a genet to persist after severe disturbance, defined as the loss of all aboveground biomass. A genet was defined as the product of a single zygote^39^. In general, we expected longevity and persistence to be higher in woody than in herbaceous plants, in resprouters than in seeders, and in clonal than in non-clonal plants^38^. Thus, we used woodiness as the first criterion in a decision tree to derive BPT (Fig. 1). As all plants that are woody aboveground are also woody belowground (but not necessarily vice versa), we particularly used belowground woodiness as a criterion. Then, in clonal plants, new ramets and their belowground organs are continuously produced. Clonal plants outlive unitary (non-clonal) plants because clonal species may replace old organs and ramets with new ones^40^. Consequently, the age of ramets is irrelevant to the age of the genet. In principle, the lifespan of clones can be very long, with estimates of several thousand years for clonal woody plants, such as *Populus eupratica*^41^ or *Jacaranda decurrens*^42^. Thus, as a definition of clonality we used the ratio of the lifespan of belowground organs to that of the genet (Fig. 1). Furthermore, and irrespective of being clonal or not, plants may have the ability to resprout from belowground organs after aboveground parts have been lost after disturbance. In this case, the lifespan of belowground parts of a plant is longer than that of aboveground parts, allowing the use of the ratio of belowground to aboveground lifespan as the ability to resprout. By combining the longevity of different compartments of plants, we defined the BPT as a categorical variable ranging from 1 to 6, corresponding to increasing belowground persistence (Fig. 1). Finally, and thus, expanding the concept of^38^, we also made use of the woodiness of aboveground organs to subdivide BPT categories 5 and 6. In biotic interactions and the ecological functions of a plant, the regenerated aboveground shoots of a resprouter present a stark contrast to between those that are herbaceous or woody. Woody parts are more long-lived and potentially can attain greater height. As a result, a resprouter that is woody aboveground (BPT 5b) might have a competitive advantage over a resprouter with herbaceous aboveground parts (BPT 5a). The same applies to clonal plants that are woody above- and belowground (BPT 6b) compared to those that are exclusively woody belowground (BPT 6a).

A plant organ was considered woody if a considerable amount of biomass was lignified. We based this information mainly on the databases included in Table 1, with additions from TRY^17^ and various other sources (Supplementary Information S1). Here, we use “woodiness” as a broad concept. According to^43,44^, “wood” in this sense is not solely formed by secondary xylem produced by a vascular cambium^45^, but also includes organs reinforced by stiff secondary or primary tissues, such as palms, bamboos, arborescent cacti and tree ferns. Lignified tissues also occur in the vascular bundles and other tissues of annual species and perennial herbs. Therefore, plants with only primary thickening were considered woody, if most of the tissues in the stem were lignified, as in palms. Plants with ordinary secondary thickening were categorized as woody, if the cells of the secondary xylem formed centripetally from the cambium were mostly lignified, which is widespread in magnoliids, eudicots and gymnosperms^45^. Then, woodiness can also occur by anomalous secondary thickening, which is characterized by producing wood by successive cambia formed in the cortex or in other tissues^46,47^. This type of wood formation is most common in woody monocots, Caryophyllales, Nyctaginaceae, Aizoaceae as well as in Gnetaceae^47,48^.

A species was considered clonal if a genet (product of one zygote) has the ability to produce or split into independent rooting units (ramets) by vegetative growth. Clonal growth in plants can occur through a variety of morphologically distinct organs (e.g. stolons, rhizomes, and roots)^49^.

A species was considered to have the ability to resprout if it was reported as being able to regrow shoots after total loss of the aboveground biomass. This delimitation was made because almost all plants can resprout after losing only a portion of their aboveground organs^44^, as occurs with herbivory or other forms of minor damage. However, the complete loss of all aboveground organs requires adaptations in the belowground compartments, as these must possess regenerative buds and a sufficient storage capacity for carbohydrates, amino acids, and other compounds to restore aboveground biomass. Non-clonal belowground organs that allow resprouting include root crowns, xylopodia and lignotubers (Table 2). Clonal plants are inherently capable of resprouting. Root crowns are stem bases located at the top of the main root (which is the category under which root crowns are listed in Table 2, also called root collars), from which some species can regrow shoots. Root crowns can occur on both herbaceous and woody roots. A xylopodium is the lignified swollen basis of a plant, either formed by the root or the hypocotyl or both^50^, from which aboveground shoots are produced. Usually, the aboveground shoots are herbaceous, but some researchers also include the production of woody aboveground shoots in the xylopodium category^45^. In this broader sense, the belowground parts of most shrubs would have equivalent functions as xylopodia, although this term is only applied to shrubs with a swollen belowground part in disturbance-prone ecosystems such as the Cerrado in Brazil^51^. Finally, lignotubers, a tuberous thickening at the base of the shoot, are formed solely by the shoot. More precisely, lignotubers are the product of the cotyledonary node and upper nodes, and do not involve any parts of the root^50^.

**Fig. 2:**
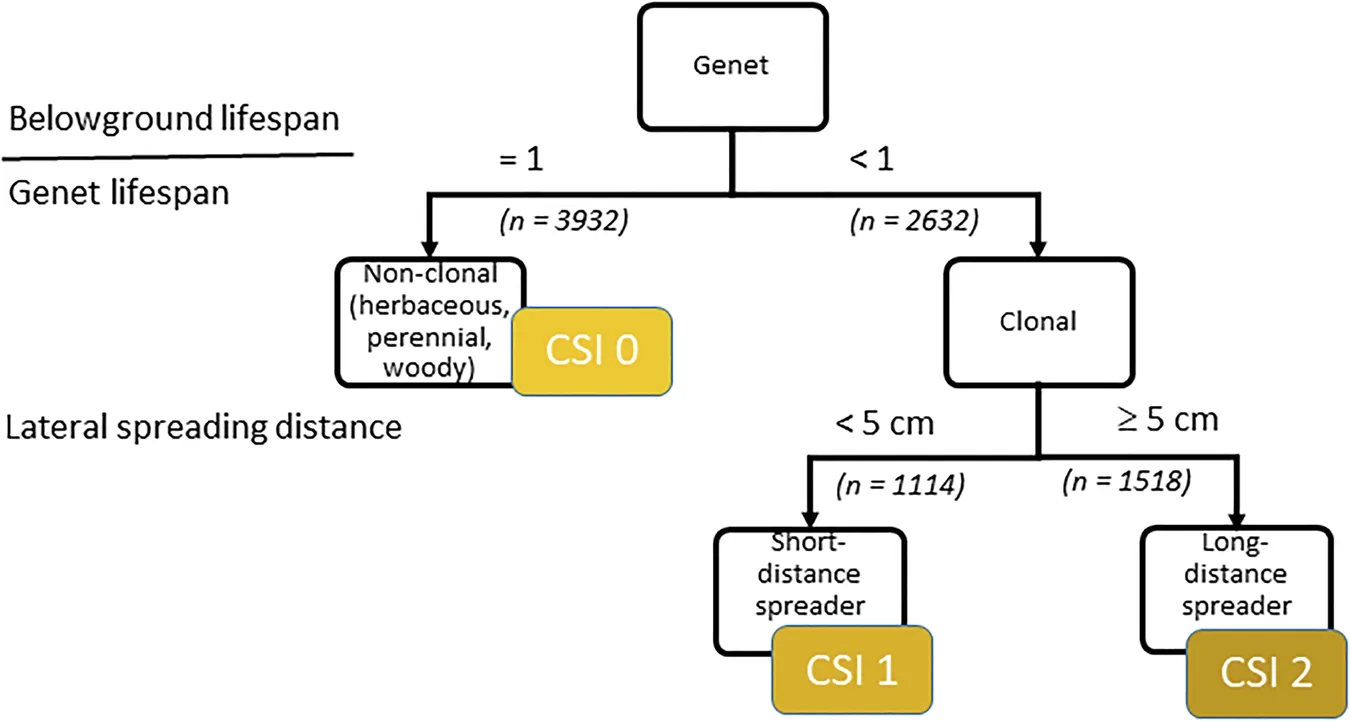
Decision tree defining the Clonal Spread Index (CSI). n shows the number of taxa in each category.

We also characterized the extent to which a clonal plant spreads horizontally by defining a Clonal Spread Index (CSI). The CSI has three ordinal levels, which designate non-clonal plants (CSI 0), plants with limited horizontal spread (CSI 1) and those that spread relatively more widely (CSI 2) (Fig. 2). As many clonal plant species are able to spread by a few centimeters, we used 5 cm as the threshold differentiating between CSI 1 and CSI 2. This threshold was mainly based on data from CLO-PLA3^16^, complemented by additional data (Table 1). For species for which the lateral spread distance was not assessed, we estimated CSI based on life form. For species with non-woody rhizomes, bulbs, and stem tubers, we assumed a short lateral spreading distance (<5 cm) and assigned them to CSI 1, whereas clonal woody species, including those with woody rhizomes, were assumed to spread wider and were categorized into CSI 2.

## Data Records

The data included in UNDERPLOT dataset as described above are available under the terms specified by CC BY 4.0 (https://doi.org/10.25829/idiv.3610-56acy9)^52^.

**Fig. 3:**
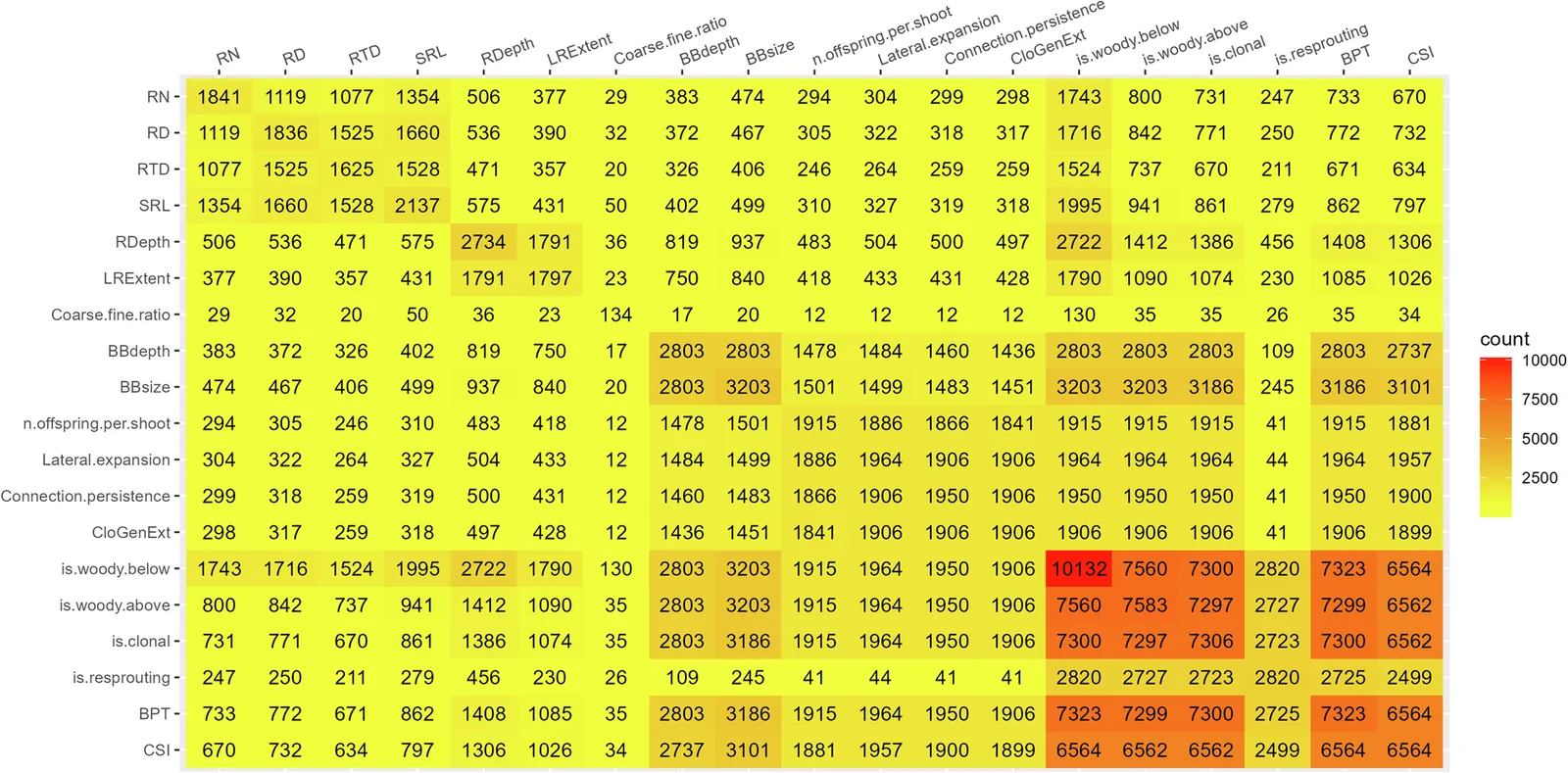
Number of species with trait values. For explanation of trait names and abbreviations, see Table 1. The figure shows the overlap in availability of trait values between pairs of belowground plant traits in the row and column. The number of species per trait is shown in the diagonal.

The number of species for which traits were available including the range in trait values is summarised in Table 1, the number of species for each category of clonal growth organs (CGOs) is found in Table 2, and the number of species for each life form is given in Table 3. In total, the UNDERPLOT dataset contains 47 fields: the aggregated species name, 21 fields related to traits, indices, growth forms and clonal growth organs (as described in Tables 1, 2 and 3), 14 fields for the number of replicates and 11 fields for original, edited, parsed and resolved taxonomic names from the different sources, for a total of 10,453 vascular plant species. The number of species with at least one trait observation differed strongly among traits, ranging between 134 (Coarse.fine.ratio) and 10,132 (is.woody.below) (Fig. 3). Table 4 shows the number of species for all combinations of BPT and CSI.

**Table 4:** Number of species by Belowground Persistence Type (BPT) and Clonal Spread Index (CSI) in the UNDERPLOT dataset.

BPT	CSI			
	0	1	2	NA
**1**	851	1	0	1
**2**	1288	1	0	10
**3**	0	1109	980	645
**4**	192	0	0	7
**5**	1	0	0	0
**5a**	352	0	16	42
**5b**	1247	0	3	36
**6a**	0	1	77	0
**6b**	1	2	442	18
**NA**	0	0	0	3130

NA = data not available.

## Data Overview

**Fig. 4:**
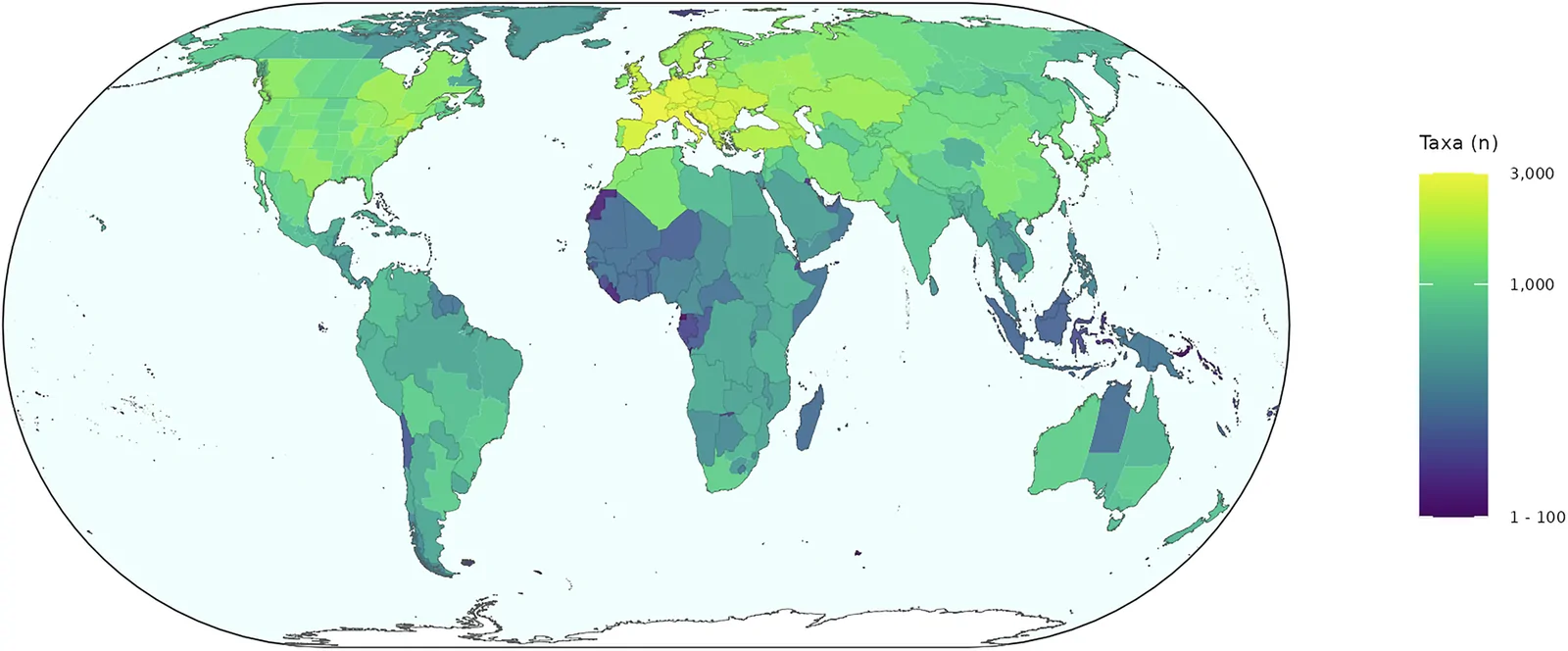
Geographical coverage of species in the UNDERPLOT dataset. Colours show the number of species by botanical country, using the information from the World Catalogue of Vascular Plants through the rWCVP R package^57^.

To illustrate the geographical extent of the dataset, we retrieved species’ distribution range for most of the species in the dataset (n = 10,298; 99%). We used the World Geographic Scheme for Recording Plant Distributions (WGSRPD)^53^ for summarizing the number of species occurring in each WGSRPD level 3 region (i.e., botanical country), which mostly corresponds to countries’ delimitations plus within-country divisions for large countries (Fig. 4). Despite our efforts to include studies from the Global South, the dataset is still heavily biased towards temperate regions. This is the result of the major databases compiled there, such as GRooT^14^, RSIP^15^ and CLO-PLA3^16^.

## Technical Validation

We validated our assessment by compiling cross-tabulations of the different trait values and trait categories to minimize the risk of inconsistencies. Taxon names were validated against the sPlot 4.1 taxonomic backbone (see code step 1 and Methods *Taxonomic standardization*). BPT and CSI values were validated using cross tables with the information on above- and belowground woodiness, resprouting capacity and clonality (see code step 4 and Methods *Definition of Belowground Persistence Types*). Inconsistencies of single species were resolved by intensive literature research (summarized in Supplementary Information S1, also done in code step 4). The last step in validation was conducted by a data curator during the data deposition in the iData repository. Please note that BPT and CSI still have numerous missing values, for which further information might be available in the literature, but which we were not able to fill in in this version.

## Usage Notes

When using the UNDERPLOT dataset, users are urged to cite the original sources of the main underlying datasets, in addition to the present paper, (sources^14,16,17,50,54–56^). As the underlying data are continuously updated, it may be useful to contact the corresponding author for the most recent versions. As described above, the dataset exhibits substantial gaps in spatial and phylogenetic representativeness. In particular, there are large information gaps for certain trait combinations (Fig. 3). Users are encouraged to help fill these gaps by providing new measurements, which the corresponding author would be happy to learn about. Future updates of the UNDERPLOT dataset will rely both on these additions and on the currently growing underlying databases. However, we note that at least one of the 10,453 vascular plant species in UNDERPLOT occurs in 2.2 million of the 2.5 million plots (88%) in the global vegetation plot database sPlot 4.1. This means that, despite this bias, data on belowground organs can provide important insights into so-far understudied functions at the level of local communities and individual species.

## Supplementary information


Supplementary Information


## Data Availability

The code for processing data from different sources, harmonizing taxon names, defining BPT and CSI values, aggregating information to single values per taxa and complementing the data with information from TRY and other sources is available on GitHub: https://github.com/idiv-biodiversity/UNDERPLOT.
